# Development of WRAP5 Peptide Complexes for Targeted Drug/Gene Co-Delivery toward Glioblastoma Therapy

**DOI:** 10.3390/pharmaceutics14102213

**Published:** 2022-10-18

**Authors:** Ana Raquel Neves, Tânia Albuquerque, Rúben Faria, Ana M. Gonçalves, Cecília Santos, Eric Vivès, Prisca Boisguérin, Luís A. Passarinha, Ângela Sousa, Diana Costa

**Affiliations:** 1 CICS-UBI—Health Sciences Research Centre, Universidade da Beira Interior, Avenida Infante D. Henrique, 6200-506 Covilhã, Portugal; 2 UCIBIO—Applied Molecular Biosciences Unit, Departamento de Química, Faculdade de Ciências e Tecnologia, Universidade NOVA de Lisboa, 2829-516 Caparica, Portugal; 3 Associate Laboratory i4HB—Institute for Health and Bioeconomy, NOVA School of Science and Technology, Universidade NOVA de Lisboa, 2819-516 Caparica, Portugal; 4 PhyMedExp, Université de Montpellier, INSERM, CNRS, 34295 Montpellier, France; 5 Laboratório de Fármaco-Toxicologia—UBIMedical, Universidade da Beira Interior, 6200-001 Covilhã, Portugal

**Keywords:** cancer therapy, cell-penetrating peptides, drug/gene co-delivery, in vitro transfection, glioblastoma therapy, targeted therapy

## Abstract

Despite the great progress over the past few decades in both the diagnosis and treatment of a great variety of human cancers, glioblastoma remains the most lethal brain tumor. In recent years, cancer gene therapy focused on non-viral vectors which emerged as a promising approach to glioblastoma treatment. Transferrin (Tf) easily penetrates brain cells of the blood–brain barrier, and its receptor is highly expressed in this barrier and glioblastoma cells. Therefore, the development of delivery systems containing Tf appears as a reliable strategy to improve their brain cells targeting ability and cellular uptake. In this work, a cell-penetrating peptide (WRAP5), bearing a Tf-targeting sequence, has been exploited to condense tumor suppressor p53-encoding plasmid DNA (pDNA) for the development of nanocomplexes. To increase the functionality of developed nanocomplexes, the drug Temozolomide (TMZ) was also incorporated into the formulations. The physicochemical properties of peptide/pDNA complexes were revealed to be dependent on the nitrogen to phosphate groups ratio and can be optimized to promote efficient cellular internalization. A confocal microscopy study showed the capacity of developed complexes for efficient glioblastoma cell transfection and consequent pDNA delivery into the nucleus, where efficient gene expression took place, followed by p53 protein production. Of promise, these peptide/pDNA complexes induced a significant decrease in the viability of glioblastoma cells. The set of data reported significantly support further in vitro research to evaluate the therapeutic potential of developed complexes against glioblastoma.

## 1. Introduction

Glioblastoma (GBM) is the most common adult brain cancer and one of the most malignant and aggressive types of tumors [1,2]. Current treatment includes surgical resection followed by radiotherapy and administration of chemotherapeutic drugs, such as Temozolomide (TMZ) and Bevacizumab [3,4,5]. The patient’s prognosis is very poor and tumor recurrence is very common [1]. This may be due to a lack of effective diagnostic approaches and a limited understanding of the early stages of tumor and glioblastoma pathology [6,7]. Furthermore, glioblastoma presents high inter- and intratumoral genetic heterogeneity, and drugs fail in passing through the blood–brain barrier (BBB) and reaching the tumor [6,7,8]. This fact instigates the search for novel therapies capable of overcoming the drawbacks of conventional treatments and providing enhanced therapeutic outcomes.

Gene therapy has emerged as a valuable therapeutic tool against serious diseases, such as cancer [9,10,11]. It consists of introducing nucleic acids into target cells, to induce a therapeutic effect [12]. Replacement of the targeted gene, gene modification (such as zinc-finger nucleases (ZFNs), Talen, or more recently CRISPR/Cas9 technology), gene augmentation, and blockage of the defective gene [12,13] are among the most explored gene-therapy approaches. Gene therapy protocols focused on the replacement of the p53 gene in tumor cells have been widely investigated in cancer therapy [14,15,16,17]. p53 is a tumor suppressor gene recognized as the guardian of the genome and it plays a critical role in maintaining genome integrity in response to various stress signals. It is also involved in numerous cellular pathways such as DNA repair, cell cycle regulation, cancer cell growth, and apoptosis [18]. It has been reported that the p53 gene is mutated in almost all human tumors [14,19,20]. The delivery of naked nucleic acids to target cells presents some drawbacks such as difficulty in entering cells and the rapid endo/lysosomal degradation after cell uptake [21]. The use of non-viral delivery systems based on polymers, lipids, dendrimers, or cell-penetrating peptides (CPPs), to facilitate their transfer to target cells, brought great advantages and is considered as a significant breakthrough for targeted gene delivery.

In this line, CPPs have been shown to have great characteristics for this purpose, due to their sequence function diversity [21]. CPPs are generally short, up to 30 amino acids, and can be classified according to their physicochemical properties and origin. Regarding their physicochemical characteristics, they are divided into cationic, hydrophobic, and amphipathic peptides [14,22,23]. Based on their origin, they can be classified into protein-derived, model, or designed CPPs. Amphipathic peptides are composed of both hydrophilic and hydrophobic domains that are responsible for the interaction with genetic content (arginine residues) and cellular internalization (tryptophan residues) [14,21,24]. In previous research, a family of tryptophan (W)- and arginine (R)-rich amphipathic peptides (WRAP) have been developed to efficiently complex nucleic acids, forming stable nano-systems [21,25]. Among those peptides, WRAP5 is a CPP with a length of 15 amino acids containing W, R, and L residues, a net positive charge (+5), and able to self-assembling into nanoparticles of 80–100 nm diameter [21,25]. It is thought that cellular transfection, mediated by WRAP5, occurs via direct translocation or endocytosis, followed by a fast endosomal escape [25]. Moreover, the grafting of targeting ligands at the surface of the nanoparticles surface can be a convenient strategy to enhance the selectivity of CPPs-based delivery vehicles [26].

One of the major obstacles in brain tumor treatment by conventional intravenous therapies is the presence of the BBB, which mechanically and biochemically limits the passage of molecules [27,28,29]. The BBB is a semipermeable and selective system in the central nervous system (CNS) that regulates the flux of ions, molecules, and cells between the blood and the brain, controlling CNS homeostasis [30,31,32]. Transferrin (Tf) is a 76-kDa blood–plasma glycoprotein responsible for ferric ion delivery (Fe^3+^) and it is known for its ability to easily penetrate brain capillary endothelial cells (BCECs) of the BBB via transferrin receptor (TfR)-mediated transcytosis. Notably, this receptor is highly expressed in the BBB and glioblastoma cells [33,34,35]. Therefore, TfR-targeting strategies can be developed for the delivery of therapeutic molecules to brain tumors [34]. Hence, several delivery systems have been functionalized with Tf as a promising strategy in the quest for suitable carriers for the treatment of this type of cancer [36,37,38].

The combination of chemotherapy and gene therapy has been demonstrated to bring improvements to cancer care, due to synergistic effects. Both in vitro and in vivo studies showed that the simultaneous release of anti-cancer drugs and therapeutic genes to cervical and gastric cancer cells led to the inhibition of tumor growth and proliferation [15,39,40]. Approaches to address glioblastoma therapy based on drug/gene co-delivery, namely on the use of TMZ combined with pDNA or siRNA, have already been demonstrated as providing enhanced therapeutic effects [41,42,43]. Despite some studies available in the literature, this chemo/gene therapy combination has been poorly explored but it should be deeply investigated for improved outcomes in glioblastoma treatment. The systemic delivery of several nano-platforms, based on natural lipids, polymers, or β-cyclodextrins, encapsulating wild-type p53, was shown to sensitize tumor cells, increase apoptosis, and reduce the tumor volume in mice [44,45,46]. Moreover, considerable progress in preclinical studies, aiming to transduce wild-type p53 to glioblastoma cells, has been achieved [47]. However, its success in the posterior phases of clinical trials has been very limited. This fact can be attributed mainly to the presence of the BBB and to the low transduction efficacy of currently available delivery systems. Another interesting perspective, in glioblastoma therapy, considers intracranial injection or injection after resection of the tumor [48,49]. For instance, nanocomplexes can be coupled to a hydrogel for deposition in the cavity left by the tumor resection for a longer drug-realize over time and to treat tumor cells that were not resected. Nanoparticles have emerged as suitable platforms for the targeted delivery of therapeutic agents to brain tumors and preclinical results are promising. Despite the efforts of scientists, there is still considerable space for the creation of new and advanced targeted delivery systems to enhance the efficacy of glioblastoma therapy. In this sense, the aim of this study was the development of a high-performance targeted pDNA/anticancer drug carrier for the co-delivery of the p53 gene and TMZ into glioblastoma cells.

In this work, we developed complexes based on WRAP5 peptide, with and without a TfR-targeting sequence, a p53 gene encoding plasmid DNA and TMZ, for glioblastoma cancer gene-therapy. The formed complexes, at several nitrogen to phosphate groups (N/P) ratios, were studied to show the complexation behavior, physicochemical properties, and pDNA stability/protection. The results showed that, according to the N/P ratio considered, peptide/pDNA complexes presented favorable characteristics for cellular uptake and transfection. Confocal microscopy confirmed the cellular internalization of these complexes into glioblastoma cells with nuclear co-localization. The cellular transfection, mediated by the conceived complexes, resulted in gene and protein expression. Moreover, the complexes were able to induce a considerable decrease in the viability of glioma cells. Altogether, these results highlight the promising therapeutic value of the developed complexes for glioblastoma treatment.

## 2. Materials and Methods

### 2.1. Materials

WRAP5 (NH_2_-LLRLLRWWWRLLRLL-CONH_2_) and Tf-WRAP5 (NH_2_-HAIYPRH-LLRLLRWWWRLLRLL-CONH_2_) syntheses were performed on a LibertyBlue™ Microwave Peptide Synthesizer (CEM Corporation, Matthews, NC, USA) with an additional Discover™ module (CEM Corporation, NC, USA) combining microwave energy at 2450 MHz to the Fmoc/tert-butyl (tBu) strategy, supplied as a lyophilized powder and kept at 4 °C until use. Peptide identity and purity were checked by LC/MS (Waters, Saint-Quentin-en-Yvelines, France), revealing a purity of ≥95%. The properties of the peptide, namely, sequence, total residues, isotopic mass, and positive charges are presented in Table S1 (Supplementary Materials).

The 6.07 kbp plasmid pcDNA3-FLAG-p53 (Addgene plasmid 10 838; Cambridge, MA, USA) was produced and purified by a procedure developed by our research group and described in the literature [50]. Temozolomide (TMZ) was acquired from Frilabo (Lisbon, Portugal), 3-(4,5-dimethylthiazol-2-yl)-2,5-diphenyltetrazolium bromide (MTT), and fluorescein isothiocyanate (FITC), isomer 1, were obtained from Sigma Aldrich Chemicals (St. Louis, MO, USA). Methanol (High Performance Liquid Chromatography (HPLC)-grade) was acquired from VWR International (Carnaxide, Portugal). Agarose and GreenSafe Premium were obtained from NZYTech (Lisbon, Portugal) and DAPI was obtained from Invitrogen (Carlsbad, CA, USA). Dulbecco’s Modified Eagle’s Medium (DMEM) high glucose with stable L-Glutamine was purchased from Biowest (Nuaillé, France) and DMEM/Nutrient Mixture F-12 Ham (DMEM/F-12) with L-glutamine cell culture medium from Sigma-Aldrich. All solutions were freshly prepared using ultra-pure grade water, purified with a Milli-Q system from Millipore (Billerica, MA, USA).

U-87 human cells, a cell line isolated from malignant glioma from a male patient, likely with Glioblastoma, were supplied by the European Collection of Authenticated Cell Cultures (ECACC, Salisbury, UK).

### 2.2. Methods

#### 2.2.1. Preparation of TMZ-Loaded Peptides

TMZ drug-loaded peptides (TMZ/WRAP5 and TMZ/Tf-WRAP5) were prepared following a previously described protocol [51]. Briefly, 150 µL of 2 mg/mL WRAP5 and Tf-WRAP5 were prepared in distilled water and 360 µL of 0.5 mg/mL TMZ ethanol solution was softly mixed and dispersed in 8 mL of water through sonication for 90 s. TMZ-loaded peptides were lyophilized overnight and then resuspended in distilled water.

The TMZ-loaded content in peptides was measured using an HPLC model Agilent 1260 system (Agilent, Santa Clara, CA, USA) equipped with an autosampler and quaternary pump coupled to an Agilent 1260 Infinity Diode Array Detector (DAD) VL (Agilent, Santa Clara, CA, USA) that delivers multiple wavelength detection with full spectra at sampling rates up to 240 Hz. The assay was performed in a Hypersil™ BDS C18 (250 × 4.6 mm × 5 μM) column following a previously defined method [52]. The mobile phase was composed of the water-methanol mixture (80:20 *v*/*v*) containing 0.5 M acetic acid, which was degassed, and vacuum filtered. The flow rate was set at 1.0 mL/min, the column temperature was adjusted to 35 °C and 20 μL of the injection volume was worked with a detection wavelength of 330 nm. A series of 5–100 µg/mL standard samples were prepared from TMZ stock solution to obtain a calibration curve. The peak area of the drug and peptide peak chromatograms were used for posterior analysis.

Drug-loaded efficiency was calculated using Equation (1):

Drug loaded efficiency (% *w*/*w*) = (Actual drug content/Initial drug content) × 100 (1)

#### 2.2.2. Formation of Peptide/pDNA Complexes

Peptides (WRAP5 and Tf-WRAP5) were suspended in water and kept at −20 °C. The final solution concentration was measured spectrophotometrically considering the absorbance at 280 nm and using a NanoPhotometer™ (Implen, Inc., Westlake Village, CA, USA). The peptide/pDNA complexes were formed by adding 50 µL of peptide solutions to 150 µL of pDNA solution (1 μg dissolved in 10 mM Tris-EDTA pH 7.0 buffer) dropwise, at vortex for 60 s. Various N/P ratios were considered for complex formation, ranging from 0.1–5, taking into account the molar ratio of positively charged amine groups (N) from the peptide to negatively charged phosphate groups (P) from pDNA. The formed complexes were left for equilibration for 25 min at room temperature. Thereafter, the complexes were centrifuged at 13,500× *g* for 20 min at 4 °C and the pellet (containing the complexes) was recovered.

To monitor the pDNA complexation capacity (CC) of the developed complexes, the supernatants from all of the complexes were evaluated by agarose gel electrophoresis. Additionally, the pDNA CC was quantified in the NanoPhotometer™ by measuring the absorbance of the supernatant at 260 nm. The pDNA CC was determined using Equation (2):

CC (%) = (Initial amount of pDNA-non-bound pDNA)/Initial amount of pDNA(2)

#### 2.2.3. Agarose Gel Electrophoresis

The agarose gel, 1% (*w*/*v*), was prepared in 50 mL 1× TAE buffer (40 mM Tris base, 20 mM acetic acid, 1 mM EDTA at pH 8.0) and stained with GreenSafe (0.6 μL). Electrophoresis ran for 40 min at 120 V and the gel was visualized with the Uvitec Fire-Reader system (Uvitec Limited, Cambridge, UK).

#### 2.2.4. Characterization of Complexes

The morphology of the peptide/pDNA complexes developed at N/P ratios of 0.5 and 1 was determined by Scanning Electron Microscopy (SEM). After formation, the complexes were centrifuged, and the obtained pellet was suspended in an aqueous solution containing 40 μL of tungsten. The solution was placed in a round-shaped coverslip and dried overnight at room temperature. An Emitech K550 (London, UK) sputter coater was used to coat the samples with gold. The complexes were visualized with a Hitachi S-2700 (Tokyo, Japan) scanning electron microscope with an accelerating voltage of 20 kV at various magnifications.

The properties of the peptide/pDNA complexes, such as the average size, polydispersity index, and zeta potential, were determined by Dynamic Light Scattering (DLS), at 25 °C. A Zetasizer Nano ZS device (Malvern Instruments, Malvern, UK) was used. For these experiments, the pellet was suspended in 5% glucose with 1 mM NaCl solution. For the size determination, a He-Ne laser at 633 nm with non-invasive backscatter (NIBS) was considered and for the surface charge, an electrophoretic light scattering optics using an M3-PALS laser (Phase Analysis Light Scattering) was employed. The obtained data were analyzed using Malvern Zetasizer software v 6.34, (Malvern Instruments, Malvern, UK).

#### 2.2.5. pDNA Protection Assay

One formulation of each of the WRAP5/pDNA and Tf-WRAP5/pDNA complexes at the different N/P ratios was incubated for 4 h with 25 μL of DMEM/Nutrient Mixture F-12 Ham (DMEM/F-12) with L-glutamine and DMEM high glucose with stable glutamine medium both supplemented with 10% FBS and streptomycin (1%)/penicillin (0.1%) antibiotics mixture solution, at 37 °C. The release and possible pDNA degradation were verified by electrophoresis, considering a 1% agarose gel. Subsequently, pDNA was decomplexed from the developed complexes by the addition of 1.5 µL of 10% SDS and incubation for 10 min. Samples were analyzed by electrophoresis for 30 min under 120 V.

#### 2.2.6. Cell Culture

U-87 human glioblastoma cancer cells were grown in 75 cm^3^ t-flasks with DMEM high glucose with stable L-glutamine medium pH 7.45, supplemented with 0.5 g/L sodium bicarbonate, 10% heat-inactivated fetal bovine serum (FBS), 1.10 g/L HEPES, and 0.1% (*v*/*v*) of the mixture constituted by penicillin (100 µg/mL) and streptomycin (100 µg/mL). Cells were kept at 37 °C, in a humidified atmosphere containing 5% of CO_2_, until confluence was achieved. Thereafter, the cells were sub-cultivated every 2/3 days to maintain their exponential growth.

#### 2.2.7. Live-Cell Imaging Assay

##### FITC Plasmid Labeling

Plasmid DNA was labeled with FITC by mixing 2 µg of pDNA, 2 μL of FITC (in sterile anhydrous dimethyl sulfoxide, 50 mg/100 µL) and labeling buffer, 81 μL (0.1 M Sodium Tetraborate, pH 8.5). Samples were left under stirring, for 4 h, at 25 °C, and in the dark. To finalize the reaction, 2.5 volumes of ethanol, 100%, (212.5 μL) and one volume of 3 M NaCl (85 μL) were added. Samples with stained pDNA were incubated at −20 °C overnight. On the following day, samples were centrifuged (10,000× *g*, at 4 °C) for 30 min. The pellet was recovered and washed with ethanol (75%) and used for the formation of peptide/pDNA complexes.

##### Cellular Uptake/Internalization

The cellular uptake of the different peptide/pDNA complexes was monitored by considering a live cell experiment and using a Zeiss LSM 710 confocal laser fluorescence microscopy (CLSM) (Carl Zeiss SMT, Inc., Oberkochen, Germany). The complexes were formed, as previously described, and considering FITC-labeled pDNA. U-87 cancer cells (10,000 cells/well) were grown in μ-slide 8-well (Ibidi, Martinsried, Germany) until approximately 50–60% confluence was attained. Then, 12 h before transfection occurred, the complete medium was replaced by FBS-free medium and without antibiotic supplementation. On the day of the acquisition, the nucleus was stained by incubating cancer cells with 1 µM DAPI for 10 min and 0.1 µg pDNA-FITC was added to each well. U-87 cells were transfected with the different complexes and images were acquired. Real live transfection was visualized during 4 h under a 63 × oil immersion objective and analyzed with the LSM software. During the acquisition, cells were maintained at 37 °C with 5% CO_2_. The images were acquired with the laser and filters corresponding to the respective DAPI (445/450 nm) and FITC (525/550 nm) dyes. The images were processed and analyzed with Zeiss Zen (blue edition) software (Carl Zeiss SMT, Inc., Oberkochen, Germany).

#### 2.2.8. Conventional Polymerase Chain Reaction (PCR)

##### Cells Transfection

U-87 cells were seeded at a density of 1 × 10^5^ cells/well onto the poly-l-lysine coverslip 12-well plate and grown in 1.5 mL of DMEM high glucose with stable L-glutamine supplemented with 10% heat-inactivated FBS and 0.1% of streptomycin/penicillin antibiotics’ mixture solution. Cells were maintained at 37 °C in a humidified atmosphere containing 5% CO_2_. At least 12 h before transfection the medium was replaced by a medium without FBS and antibiotics, to promote transfection. On the day of the experiment, cells were transfected with the complexes (1 µg of pDNA/well) and incubated for 4 h. Subsequently, cells were returned to their usual culture medium and collected after 24 h.

##### cDNA Synthesis and PCR

The culture medium was removed, and cells were washed with phosphate buffer solution (PBS). Untreated cells were used as the control. To collect and extract total RNA, tripleXtrator (GRiSP, Porto, Portugal) (250 μL) was added to the cells and incubated at room temperature for 5 min, followed by the addition of 50 µL of chloroform and vigorous stirring, according to instructions provided by the manufacturer. The obtained samples were quantified by using a NanoPhotometer™ and additionally they were run on an agarose gel (1%) and analyzed by electrophoresis. The cDNA synthesis was performed by using the “Xpert cDNA Synthesis Kit” from GRiSP (GRiSP, Porto, Portugal), following the manufacturer´s protocol. PCR amplification of p53 cDNA was performed by using the Speedy Supreme NZYTaq 2× Green Master Mix (NZYTech, Lisbon, Portugal), following the manufacturer´s protocol. In each PCR reaction, 15 µL of nuclease-free water, 2.5 μL of primer reverse (5′-CTG AGT CAG GCC CTT CTG TCTT-3′), and primer forward (5′−GAG CTG AAT GAG GCC TTG GA-3′) diluted in 1:20, 25 μL of Speedy Supreme NZYTaq, 2 × Green Master Mix, and 5 μL of cDNA were added. Samples were then placed in a T100™ Thermal Cycler (Bio-Rad Laboratories, Inc., Hercules, CA, USA), and the following conditions were considered: initial denaturation (95 °C for 5 min); denaturation (94 °C for 2 s); annealing (53 °C for 5 s); extension (72 °C for 1 min) for 29 cycles; and a final extension (72 °C for 2 min). PCR products were analyzed by electrophoresis on an agarose gel and visualized in the Uvitec Fire-Reader system.

#### 2.2.9. p53 Protein Quantification

The cells were transfected following the procedure mentioned above. The medium was removed, and U-87 cells were washed (3 times) with phosphate buffer solution (PBS). Cells were then collected and the p53 content was quantified by using the p53 pan ELISA kit (Roche Applied Science, Penzberg, Germany). This kit is based on a sandwich enzyme-immunoassay. All of the experimental protocol steps, provided by the manufacturer, were followed. The levels of p53 were spectrophotometrically determined, at 450 nm, using a Shimadzu UV–vis 1700 spectrophotometer. Non-transfected cells and cells transfected with naked pDNA were considered as controls.

#### 2.2.10. Cytotoxicity Study

The cytotoxicity of the developed peptide/pDNA complexes was evaluated on U-87 cells using the MTT (3-[4,5-dimethyl-thiazol-2-yl]-2,5-diphenyltetrazolium bromide) assay. Cells were seeded in 96-well plates at a density of 1 × 10^4^ cells/well and grown at 37 °C in a 95% air/5% CO_2_ humidified atmosphere. The different peptide/pDNA complexes (100 µL) were resuspended in serum-free DMEM medium and applied to the well plates for 6 h. To finish the transfection, the medium was changed. The MTT reduction was evaluated after incubation for 24 h or 48 h. After incubation, the culture medium was discarded and 200 µL of dimethyl sulfoxide (DMSO) was added to each well. The plate was placed to shake for 30 min, protected from light, to dissolve the formazan crystals. Thereafter, the redox activity was monitored through MTT reduction by measuring the absorbance at 570 nm using a Benchmark Microplate Reader (BioRad, Vienna, Austria). The medium without cells was settled as zero absorbance and used for spectrophotometer calibration. Non-transfected cells and ethanol-treated cells were considered to be the positive and negative controls, respectively. Additionally, cells transfected with naked pDNA were also considered as control. The relative cell viability (%) related to control wells was calculated by [A]_test_/[A]_control_ × 100, where [A]_test_ is the absorbance of the test sample and [A]_control_ is the absorbance of the positive control sample.

#### 2.2.11. Statistical Analysis

The normality of the distribution of sample data was analyzed by performing normality tests, namely D’Agostino and Pearson omnibus and Kolmogorov–Smirnov tests. The statistical analysis considered a *t*-test and one-way analysis of variance (ANOVA) followed by the Bonferroni test. All quantitative data are expressed as the mean ± SEM of at least three measurements. All data results were analyzed using GraphPad Prisma V9.0.0 software. A *p*-value below 0.05 was considered statistically significant. Additionally: *, *p* ≤ 0.05; **, *p* ≤ 0.01; ***, *p* ≤ 0.001; ****, *p* ≤ 0.0001.

## 3. Results and Discussion

### 3.1. TMZ Loading Efficiency

Pursuing the goal of developing a suitable and efficient delivery system for glioblastoma therapy, the WRAP5 peptide bearing the Tf targeting sequence was considered to condense a p53 encoding pDNA and form nanocomplexes. To increase the functionality of the developed nanocomplexes, the anticancer drug TMZ was also loaded into these complexes. TMZ was incorporated into WRAP5 peptides by following the procedure described in the experimental section. According to published data, TMZ may interact with amino and carboxyl termini at the side chains of amino acids of peptides [53].

It is crucial to use an appropriate methodology for TMZ determination with accurate quantification. To evaluate the amount of TMZ-loaded peptide, an HPLC method was applied (HPLC-DAD). To assess the linearity of the method, a representative curve was plotted (Figure S1). The calibration curve was obtained by plotting the peak area ratio between the analyte versus the analyte concentration [28]. Each calibration point results from triplicates and was plotted as Mn ± SD resulting in a correlation coefficient (r^2^) higher than 0.99 (Figure S1).

**Figure 1:**
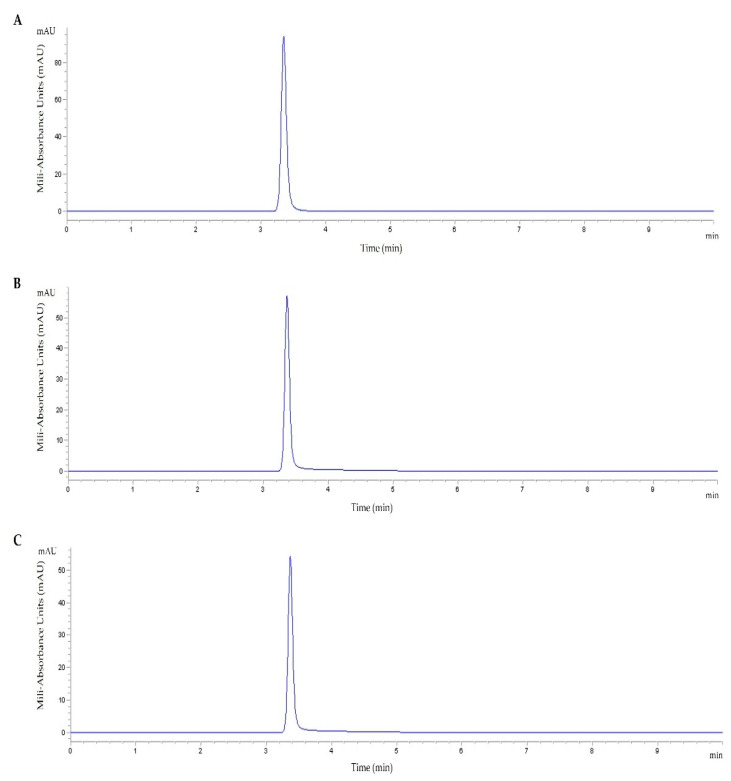
HPLC chromatogram for TMZ standard sample at 20 µg/mL (**A**), TMZ/WRAP5 at 20 µg/mL (**B**) and TMZ/Tf-WRAP5 at 20 µg/mL (**C**); retention time (TMZ) of 3.4 min.

HPLC chromatograms of the TMZ standard sample and TMZ-loaded peptide solutions, shown in Figure 1, proved that the method provides an excellent resolution of TMZ without interfering molecules. The run time was safely set to 10 min, to ensure no subsequent and sequential contamination of the samples resulting from the formulations under study. A typical chromatogram of TMZ drugs is shown in Figure 1A. As can be seen in the chromatogram of TMZ/WRAP5 (Figure 1B) and TMZ/Tf-WRPA5 (Figure 1C), a specific peak corresponding to TMZ with the same retention time as the peak of the TMZ standard sample (retention time of 3.4 min) was detected. Moreover, it was confirmed that the peptide does not interact with the C18 column since its presence was not observed under the isocratic operation conditions tested. Therefore, this ensures TMZ detection. These results demonstrated TMZ loading into both WRAP5 and Tf-WRAP5 peptides. Considering the initial amount of TMZ and the actual amount of TMZ present in the peptide solution, TMZ-loaded efficiency was estimated. The obtained values are presented in Table 1. These results correlated well with documented data [51,54].

### 3.2. pDNA Complexation

**Figure 2:**
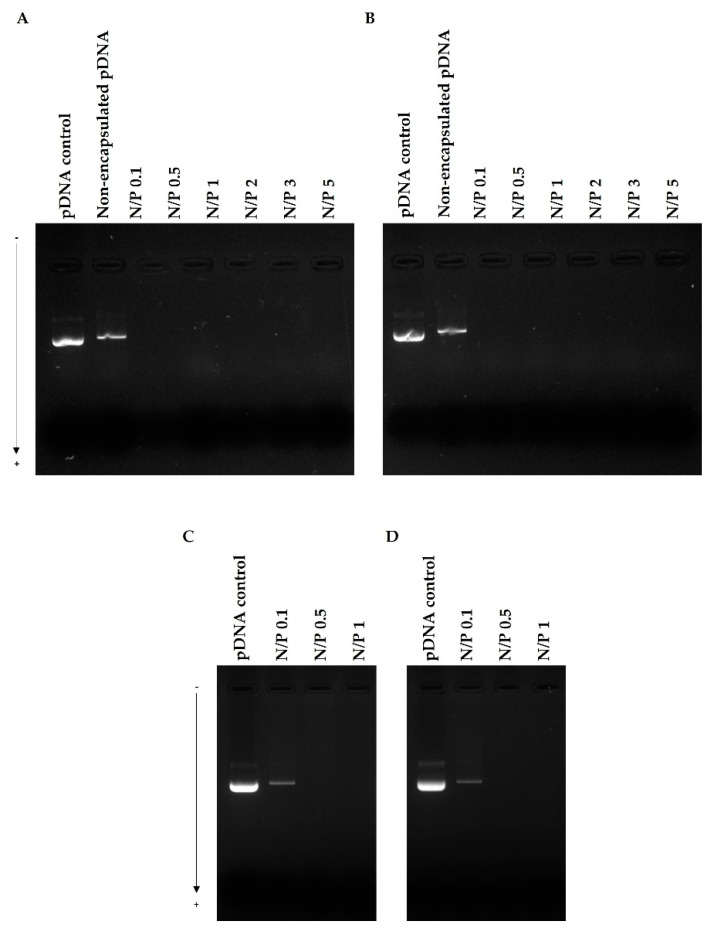
WRAP5/pDNA (**A**), Tf-WRAP5/pDNA (**B**), TMZ/WRAP5/pDNA (**C**), and TMZ/Tf-WRAP5/pDNA (**D**) complexation behavior at various N/P ratios investigated by agarose gel electrophoresis. The complexes were formulated, centrifugated, and the supernatants containing the potential free pDNA were loaded on the agarose gel (See Scheme S1A). Lane 1: pDNA control sample.

The capacity of WRAP5, Tf-WRAP5, TMZ/WRAP5, and TMZ/Tf-WRAP5 to complex pDNA was investigated, at different N/P ratios (ranging from 0.1 to 5). As described in the experimental section, after centrifugation of the developed complexes, the supernatants were evaluated by agarose gel electrophoresis to detect the presence of free pDNA and, therefore, determine the pDNA CC displayed by the complexes. The experimental procedure is summarized in Scheme S1A (Supplementary Materials). The results are presented in Figure 2. The pDNA-based complexes were formed by a combination of electrostatic and hydrophobic interactions with the consequent formation of nanoparticles [50,55,56]. The arginine residues of WRAP5 mainly contributed to the electrostatic interaction with pDNA while tryptophan residues, most probably, interacted with the minor groove of pDNA by hydrophobic interactions [21].

The N/P ratio is demonstrated to deeply influence the complexation behavior and, therefore, it was identified as a tailoring parameter [50,57]. As visualized in Figure 2, WRAP5 and Tf-WRAP5 peptides could efficiently complex pDNA at N/P ratios higher than 0.1. From this ratio, peptides were able to complex pDNA, neutralizing its charges, and, therefore, pDNA could not migrate through the agarose gel. The guanidinium and amine groups from the WRAP5 peptide strongly interact with pDNA negatively charged phosphate groups, originating pDNA-based complexes. The presence of the targeting sequence Tf which contains an additional positive charge did not seem to influence the peptide capacity for pDNA complexation, as the same pDNA complexation pattern with N/P ratio was observed (Figure 2B). The incorporation of TMZ into peptide/pDNA complexes also kept this profile, as demonstrated in Figure 2C,D. TMZ/WRAP5 and TMZ/Tf-WRAP5 peptides were both able to promote efficient pDNA complexation, at least, at N/P ratios from 0.5, since no bands were visualized in the agarose gel. This study confirmed the great capacity of the WRAP5 peptide to complex pDNA (1 µg), at ratios above 0.1, and in line with this and for subsequent in vitro investigations, there was no need to formulate peptide/pDNA complexes at higher N/P ratios (N/P of 2, 3 or 5).

### 3.3. Physicochemical Properties of the Complexes

**Figure 3:**
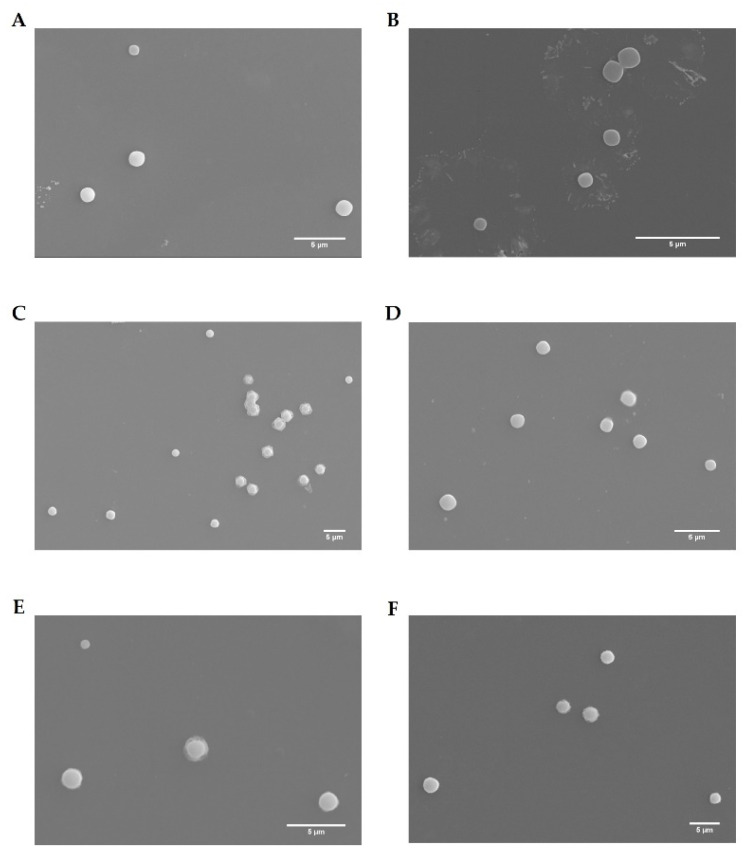
Scanning electron micrographs of WRPA5/pDNA complexes at N/P ratios of 0.5 (**A**) and 1 (**B**), Tf-WRAP5/pDNA complexes at N/P ratios of 0.5 (**C**) and 1 (**D**), and TMZ/WRAP5/pDNA (**E**) and TMZ/Tf-WRAP5/pDNA (**F**) complexes both prepared at N/P ratio of 0.5. Scale bar = 5 µm.

The morphology, mean size, polydispersity, surface charge, and pDNA complexation capacity (CC) of the developed complexes (WRAP5/pDNA, Tf-WRAP5/pDNA, TMZ/WRAP5/pDNA, and TMZ/Tf-WRAP5/pDNA) were investigated as follows. The morphology of the various complexes was assessed by SEM. The obtained images are presented in Figure 3. All formed complexes exhibited a spherical shape and an apparently smooth surface. From this, it is expected that spherical peptide/pDNA complexes can be easily internalized into cells. As widely recognized, the shape may have a great influence on the success of cellular uptake/internalization of delivery systems. Spherical or semispherical nanoparticles easily interacted with the cellular membrane, enhancing the rate of cellular uptake [58,59].

Further analysis of the properties displayed by the complexes, at different N/P ratios (0.1, 0.5, and 1), was performed by DLS. The results are presented in Table 2. All formed complexes had sizes below 500 nm, and this parameter varied with the N/P ratio considered at the complex formation step. As observed, lower-sized complexes were obtained with an increase in the N/P ratio. The higher amine content strengthens the interaction between WRAP5 and pDNA, increasing pDNA condensation and, therefore, leading to smaller complexes [14,17,57]. This trend was observed for all peptide/pDNA complexes and the presence of both the Tf sequence and TMZ did not seem to affect it. Moreover, the lower size complexes were the ones based on TMZ/WRAP5/pDNA and TMZ/Tf-WRAP5/pDNA developed at an N/P ratio of 1. The incorporation of TMZ contributed to a reduction in the size of the complexes, probably due to strong hydrophobic interactions established with the WRAP5 peptide leading to additional compaction. Moreover, the N/P ratio can be explored to tailor the size of developed complexes. At this point, it was also relevant to analyze the polydispersity index (PdI) of the samples, as a measure of size distribution. As documented, polydisperse systems present values from 0.4–0.7, while the monodisperse ones show PdI values from 0.01 to 0.4. A broad particle size distribution is characterized by PdI higher than 0.7 [60]. Apart from WRAP5/pDNA at N/P ratio of 0.1, Tf-WRAP5/pDNA at N/P of 0.1, and TMZ/Tf-WRAP5/pDNA at N/P of 1, all of the complexes under study presented PdI values below 0.4. Thus, we assumed that the most formed peptide/pDNA delivery systems are monodisperse, although we are aware that it was desirable to achieve lower PdI values.

The surface charges of the complexes were also determined. The formulated complexes displayed positive charges for all N/P ratios considered, and this parameter seemed to influence the extent of this positive charge. The highest zeta potential values were found for peptide/pDNA complexes prepared at high N/P ratios, namely N/P of 1. This effect was more pronounced for TMZ/WRAP5/pDNA and TMZ/Tf-WRAP5/pDNA complexes. These complexes presented zeta potential values above +10 mV when conceived at an N/P ratio of 1. Once more, this fact may directly correlate with the high amount of positive cationic charges from the peptide. Moreover, as TMZ displays no charges, a clear explanation for the surface charge increase observed for TMZ/peptide/pDNA complexes cannot be anticipated at this stage.

The positive charges exhibited by the complexes may certainly facilitate their cellular internalization, as complexes may strongly interact with anionic-sulfated proteoglycan molecules present at the cell surface. This is the main reason explaining the efficient cellular uptake of the most positively charged particles, over the ones bearing a net negative charge [61,62]. However, for some cells, such as phagocytic cells, the effect can be different, and selective cell internalization of anionic nanoparticles may occur [63].

As reported, cellular uptake/internalization is a phenomenon influenced by the set of physicochemical characteristics displayed by the delivery system [59,61,62,64]. Properties such as the geometry or shape, size, surface charge, surface chemistry, or ligand binding are known to exert a critical role in the intracellular delivery of a carrier. In general, spherical delivery systems with sizes around 100–200 nm and positively charged surfaces are preferentially captured by cells, most probably through clathrin- or caveolin-mediated endocytosis [65,66]. Cancer cells possess overexpressed receptors at their surface allowing specific ligands to bind and mediate the endocytosis mechanism (receptor-mediated endocytosis). In glioblastoma cells, TfR plays an important role in the transcytosis process [33,36].

The pDNA complexation capacity ((CC), %) of the developed peptide/pDNA complexes was also determined by Equation (2). Data are shown in Table 2. The formulations were able to complex pDNA to an extent dependent on the N/P ratio. For peptide/pDNA complexes at an N/P ratio of 0.1, less than 60% of pDNA was complexed. For all complexes, this percentage significantly increased when a higher N/P ratio was considered. At an N/P ratio of 1, and depending on the system, pDNA CCs were between 89–95%, demonstrating the efficacy of WRAP5 for pDNA complexation. As mentioned before, an increase in cationic content led to a strong electrostatic interaction with pDNA, compacting it to a larger extent, which consequently increased pDNA CC. The obtained pDNA CC values agreed well with the agarose gel electrophoresis study presented above (Figure 2).

### 3.4. pDNA Protection Assay

**Figure 4:**
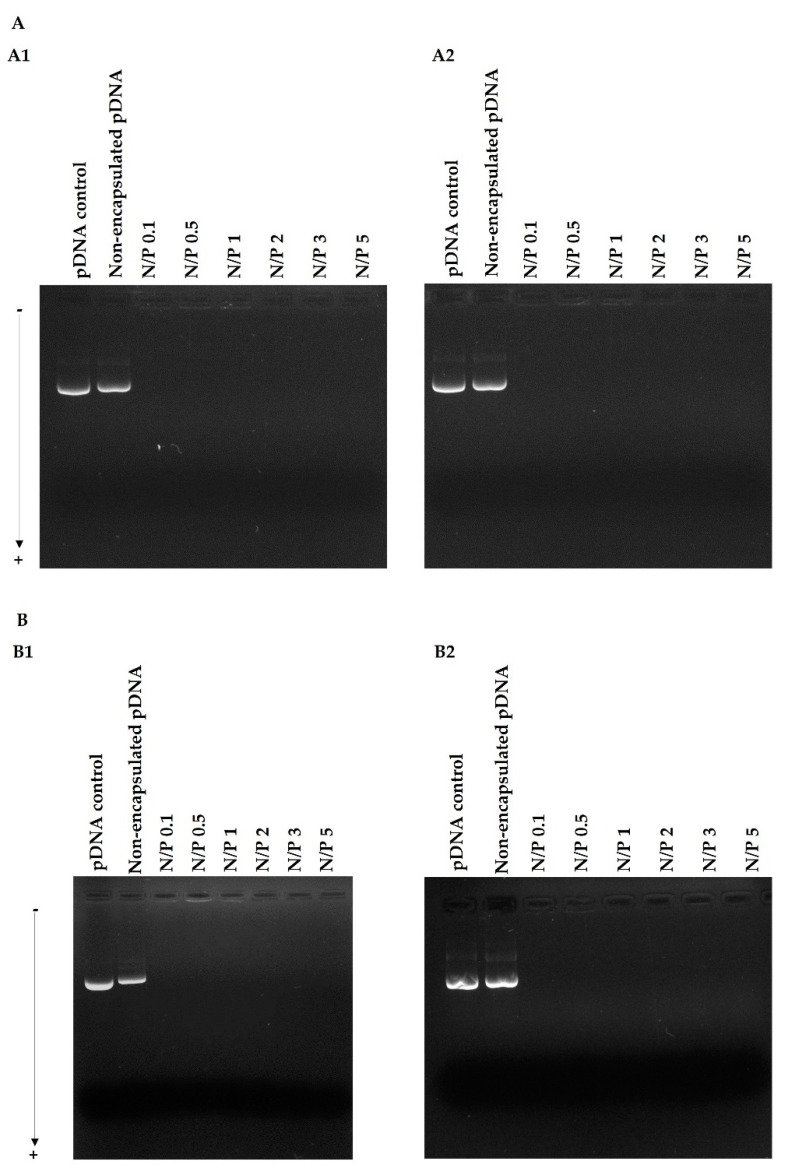
Electrophoretic analysis of WRAP5/pDNA (**A**) and Tf-WRAP5/pDNA complexes (**B**) formulated at different ratios after 4 h of incubation with DMEM/Nutrient Mixture F-12 Ham (DMEM/F-12) with L-glutamine (1) and DMEM high glucose with stable glutamine medium (2). Lane 1: pDNA control and lane 2: non-encapsulated pDNA. Complexes were loaded on the agarose gel without a centrifugation step (see Scheme S1B).

To evaluate the peptide/pDNA complex stability in the extracellular compartment and the protection they confer to pDNA, WRAP5/pDNA, and Tf-WRAP5/pDNA, complexes at different N/P ratios were incubated for 4 h with DMEM/F-12 with L-glutamine and DMEM high glucose with stable glutamine medium, at 37 °C. The results are summarized in Figure 4A,B, for WRAP5/pDNA and Tf-WRAP5/pDNA complexes, respectively. The electrophoretic migration of complexes prepared at various N/P ratios revealed the absence of free pDNA. The electrophoretic study also demonstrated that both WRAP5/pDNA and Tf-WRAP5/pDNA complexes were able to maintain pDNA integrity, at least for 4 h incubation with the above mentioned media. This observation suggested that the formed peptide/pDNA complexes are stable and ensure pDNA protection.

This asset displayed by peptide/pDNA complexes is particularly relevant as the stability of payload delivery systems is a critical issue for efficient in vitro and in vivo cellular transfection, since it can compromise payload protection, transport, expression level, and, ultimately, therapeutic response [67].

At this stage, we are fully aware that the complexed pDNA should be visible as a larger band or even a complex that does not leave the well. As no other explanation is reasonable, we believe that the pDNA complexation degree by the peptide is so high that possibly prevents strong interaction with the dye, and thus pDNA is not efficiently stained and therefore not visible in the gel wells. pDNA is surrounded by the peptide, making interactions with the dye not possible. We confirmed this by performing the gel-shift assay, without the centrifugation step, for all complexes at an N/P ratio of 1; for the results please consult Scheme S1B. The nanoparticles were not visible in the gel wells (Figure S2). To unequivocally show the presence of the complexes, in another experiment and after incubation of the complexes with the above-mentioned culture media, SDS was added to promote the de-complexation of pDNA from the complexes. The results are presented in Figure S3. In the presence of SDS, pDNA can efficiently be released from the peptide complexes. Moreover, pDNA keeps its integrity and supercoiled isoform after being released. This pDNA isoform is the most bioactive and the one that promotes higher levels of p53 gene expression. Therefore, the developed complexes may be suitable delivery systems for gene release in vivo.

### 3.5. pDNA Cellular Internalization

Our results seemed to prove that the complexes developed at an N/P ratio of 1 exhibited the most suitable properties, such as low size, a positively charged surface, and higher pDNA CC for cell entry, internalization, and pDNA accumulation. Thus, there is no advantage in using ratios beyond N/P of 1. Following this, the ability displayed by the developed WRAP5/pDNA complexes, at this N/P ratio, for cellular uptake and intracellular co-localization was investigated by fluorescence confocal microscopy in U-87 cells.

**Figure 5:**
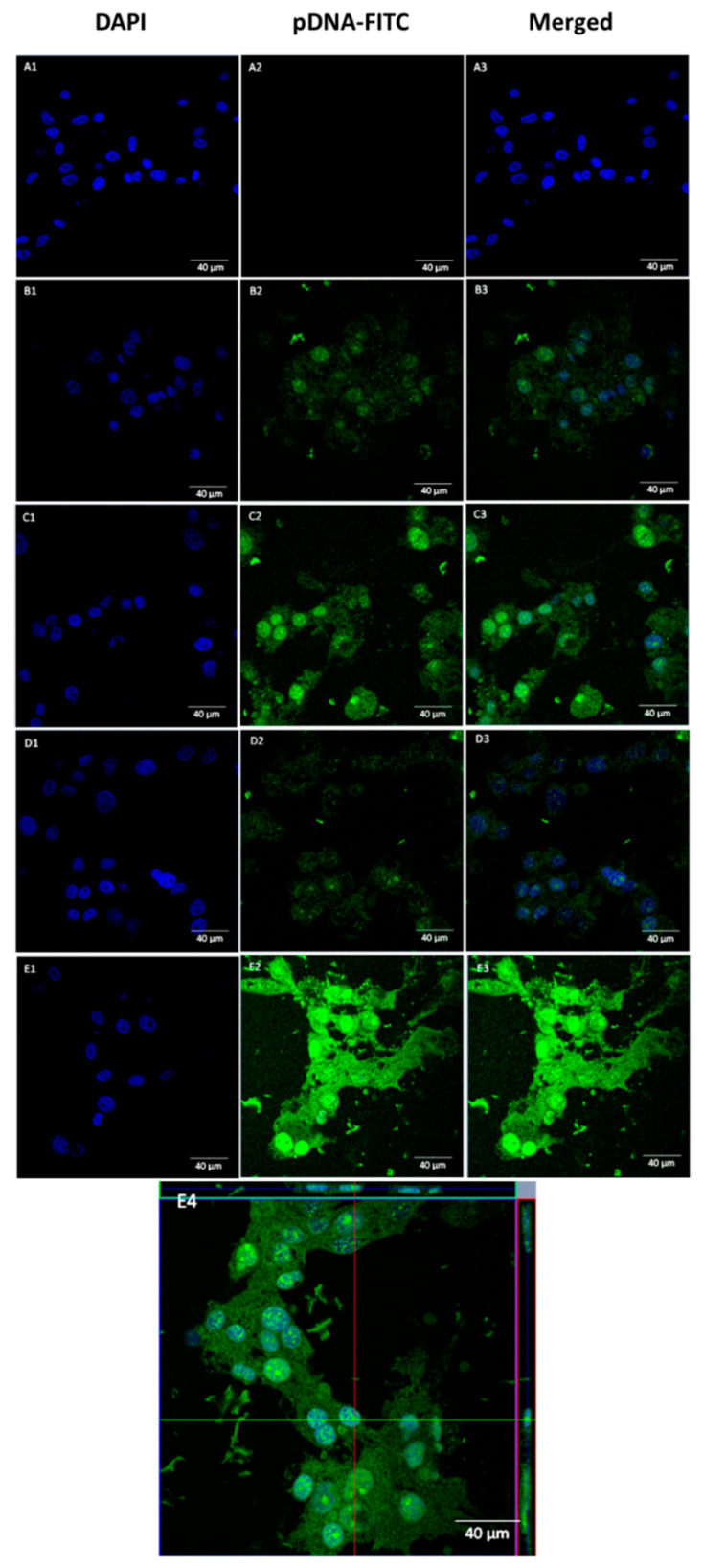
Fluorescence confocal microscopy evaluation of cellular uptake and intracellular co-localization displayed by the set of complexes, prepared at an N/P ratio of 1, after 4 h of transfection. Nuclei were stained blue by DAPI and green represents pDNA stained with FITC. The first panels show nuclei stained by DAPI, the second panels indicate labeled pDNA-FITC and the third panels correspond to merged images. Representative live-cell images of non-transfected U-87 cells (**A**), and U-87 cells transfected with the following complexes: WRAP5/pDNA (**B**), Tf-WRAP5/pDNA (**C**), TMZ/WRAP5/pDNA (**D**), TMZ/Tf-WRAP5/pDNA (**E**), and TMZ/Tf-WRAP5/pDNA, orthogonal view (E4). Scale bar = 40 μm.

A live cell experiment was performed at 4 h of transfection. Images were collected from a series of consecutive Z planes (Z-stacks, step size of 0.1 µm). Microscopy images of U-87 cells are presented in Figure 5. The nuclei were stained by DAPI and pDNA was labeled by FITC. As presented in Figure 5, the green signal from pDNA was absent in non-transfected cells (Figure 5(A1–A3)). Effective transfection with WRAP5/pDNA complexes revealed pDNA co-localization with the nucleus (Figure 5(B1–B3)). The same observation, and to a higher extent, was made when U-87 cells were transfected with Tf-WRAP5/pDNA complexes (Figure 5(C1–C3)). The presence of the Tf sequence seemed to increase transfection efficiency, as high-intensity pDNA green fluorescence was visualized. Tf-WRAP5/pDNA complexes seemed to display a high ability to target U-87 cells and reach the nucleus. Image D in Figure 5 proved the successful transfection mediated by TMZ/WRAP5/pDNA complexes, and pDNA co-localization with the nucleus, as evidenced in the merged image (Figure 5(D3)). Finally, transfection with TMZ/Tf-WRAP5/pDNA was also demonstrated to be efficient. As observed in Figure 5(E3), stained pDNA was able to be internalized and reached the nucleus. The orthogonal view of U-87 cells, after transfection mediated by TMZ/Tf-WRAP5/pDNA complexes, exhibited in image at Figure 5(E4), strongly supported the evidence of the presence of labeled pDNA in the nucleus.

This confocal microscopy study revealed that the set of developed peptide/pDNA complexes (N/P ratio of 1) was internalized into U-87 cells, transposed both extracellular and intracellular barriers, and, therefore, pDNA was able to reach the nucleus. Once this cellular organelle is reached, it is expected that p53 gene transcription and subsequent p53 protein expression take place.

As desired, the Tf sequence enhances cell entry and pDNA accumulation into the nucleus. Since transferrin receptors are upregulated in glioblastoma cells, the use of transferrin reveals itself as suitable to promote targeted cellular uptake/transfection. Several studies demonstrated that this strategy is a successful means of highly effective cell-targeting in cancer therapeutics [68,69,70].

The efficiency of cellular transfection mediated by developed WRAP5/pDNA complexes appeared to be dependent on transfection time (Figure S4). The microscopy study performed on U-87 cells after 2 h of transfection with the various complexes showed less efficacy and poor or inexistent pDNA in the nucleus. Therefore, the time of transfection can be a relevant variable to tailor/optimize the efficiency of transfection and, consequently, pDNA intracellular levels.

### 3.6. p53 Gene and Protein Expression

**Figure 6:**
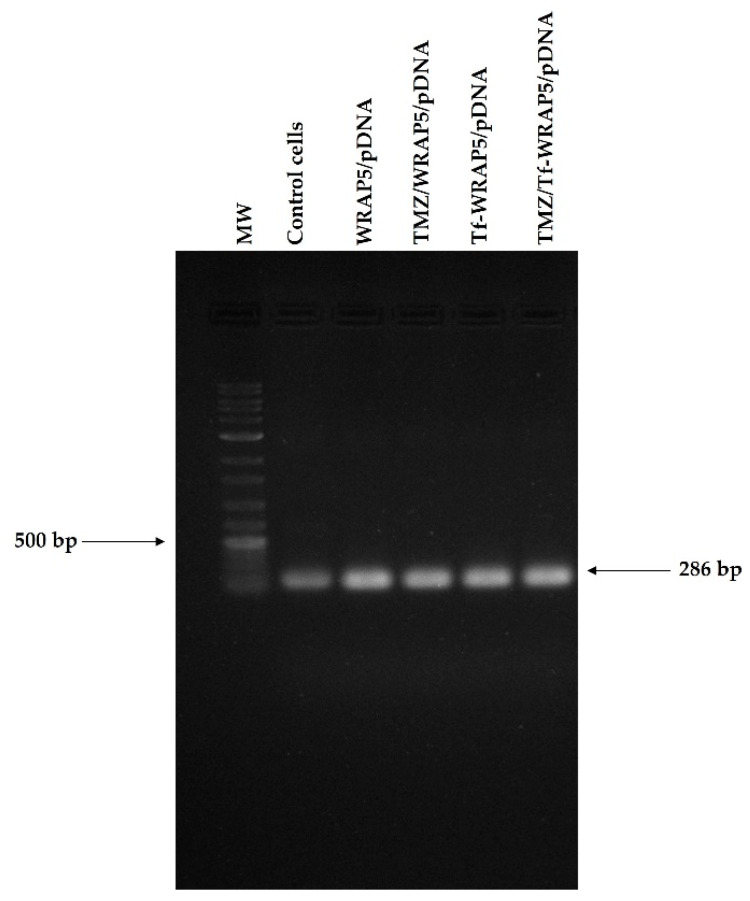
PCR analysis of p53 mRNA in U-87 cells after 24 h of transfection mediated by the developed complexes formed at an N/P ratio of 1. MW-DNA ladder molecular weight marker.

From the evidence of cellular internalization, with nucleus co-localization, of the developed peptide/pDNA complexes, p53 mRNA expression was evaluated at 24 h by PCR. Non-transfected cells were considered as the control. The obtained data can be seen in Figure 6. This figure shows an agarose gel electrophoresis of amplification products of the p53 gene. In comparison with the control, which presents a faint band, wide and intense bands were observed for all complexes studied that indicated higher levels of mRNA transcripts. The intensity of the bands was comparable among the various complexes. All developed peptide/pDNA complexes seemed to be able to promote efficient p53 gene expression in U-87 cells. However, although the PCR assay provides qualitative analysis and a more accurate methodology is necessary to deeply evaluate gene expression, it strongly suggested efficient cellular transfection followed by gene expression.

**Figure 7:**
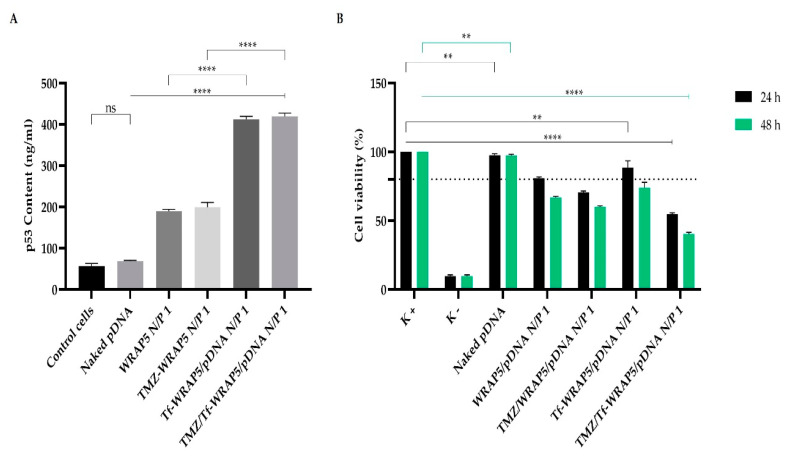
(**A**) Quantification of p53 protein levels (ng/mL) in U-87 cells after 48 h of transfection mediated by the different complexes developed at an N/P ratio of 1. The values were calculated with the data obtained from three independent measurements (mean ± SD, *n* = 3) and analyzed by one-way ANOVA with the Bonferroni test. For all complexes, ****, *p* < 0.0001 relatively to control cells. (**B**) Cellular viability of U-87 cells after 24 h and 48 h of incubation with WRAP5/pDNA, TMZ/WRAP5/pDNA, Tf-WRAP5/pDNA and TMZ/Tf-WRAP5/pDNA complexes prepared at N/P ratio of 1. Non-transfected cells were used as a positive control (K+) and cells treated with ethanol were used as negative control (K−). Statistical analysis was completed using one-way ANOVA with data obtained from four independent measurements (mean ± SD, *n* = 4). (****, *p* ≤ 0.0001; **, *p* ≤ 0.01). ns—statistically non-significant.

Furthermore, protein expression was quantified in U-87 cells, after 48 h of transfection with the different peptide/pDNA complexes, through a p53 ELISA immunoassay. The p53 amount obtained for each delivery system investigated is presented in Figure 7A.

In contrast, p53 protein production after cellular transfection with the different complexes was evaluated at 48 h as the process of mRNA transduction and protein translation is sequential [14,71,72]. A comparison with non-transfected cells (control) is statistically significant (for all complexes, *p* < 0.0001), except for the transfection of cells with only pDNA (naked), statistically non-significant. This demonstrated the relevance of pDNA complexation in nano-systems for efficient cellular transfection, and thus, protein expression. The extent of p53 content considerably varied between the complexes formulated with WRAP5 and Tf-WRAP5. A high p53 content was quantified after transfection of cells with Tf-WRAP5/pDNA and TMZ/Tf-WRAP5/pDNA complexes, *p* < 0.0001 for WRAP5/pDNA versus Tf-WRAP5/pDNA, and TMZ/WRAP5/pDNA versus TMZ/Tf-WRAP5/pDNA. From our data, it became clear that the Tf sequence positively influenced cellular transfection, consequently contributing to higher p53 production. This result agreed with the findings from the confocal microscopy study, where a strong fluorescence was observed for the complexes formulated with Tf-WRAP5. These complexes, therefore, seemed to be suitable to promote p53-based cancer therapy. Moreover, the presence of TMZ did not influence the p53 levels obtained. No statistically significant differences were found between the complexes in the absence of TMZ and the ones where this drug was incorporated.

### 3.7. Cytotoxic Effect

Information on the cytotoxic effect, promoted by the developed complexes at an N/P ratio of 1 on U-87 cells, at 24 h and 48 h, was assessed by the MTT assay. Non-transfected cells were considered as a positive control for cellular viability, while cells treated with ethanol were used as a negative control. For comparison purposes, results from cells transfected with naked pDNA are also shown; at 24 h and 48 h, ** *p* < 0.01 for the comparison with the positive control. Figure 7B summarizes the obtained results. For all studied complexes, the viability of U-87 cells decreased with the time of incubation; at 48 h, the extent of viable cells was lower compared to the one found at 24 h. Furthermore, at 24 h and 48 h, the incorporation of TMZ into the complexes enhanced the capacity of the complexes to inhibit cellular viability. The comparison between WRAP5/pDNA and TMZ/WRAP5/pDNA complexes and the one between Tf-WRAP5/pDNA and TMZ/Tf-WRAP5/pDNA complexes was statistically significant (at 48 h, ** *p* < 0.01 for WRAP5/pDNA versus TMZ/WRAP5/pDNA and **** *p* < 0.0001 for Tf-WRAP5/pDNA versus TMZ/Tf-WRAP5/pDNA). As U-87 are wild type for the p53 protein, the p53 supplementation does not lead to a strong effect on cellular viability and the main effect on this parameter is achieved by the action of TMZ. To perceive completely the effect of p53-based anticancer therapy, studies on mutated p53 glioblastoma cell lines should be performed. We intend to realize them in a near future.

Data also revealed the role played by the Tf sequence on WRAP5. The comparison between TMZ/WRAP5/pDNA and TMZ/Tf-WRAP5/pDNA complexes showed statistically significant differences: at 48 h, **** *p* < 0.0001 for TMZ/WRAP5/pDNA versus TMZ/Tf-WRAP5/pDNA complexes. This result seemed to indicate that mechanisms such as glioma cell targeting, internalization, and gene/protein expression were improved when TMZ/Tf-WRAP5/pDNA complexes mediated cellular transfection. These complexes were the ones inducing the lowest cellular viability at 48 h (approximately 40%). This fact is related to the physico-chemical properties displayed by the complexes, which facilitated their targeting and cellular uptake, therefore, mainly increasing the action of TMZ, which has direct consequences on cellular viability.

The cytotoxic effect induced by the complexes also seemed to vary with the N/P ratio, as shown in Figure S5. For the same type of complex, as the N/P ratio increased, the cell viability decreased. For instance, at 48 h, Tf-WRAP5/pDNA formulated at an N/P ratio of 0.1 was biocompatible in relation to the positive control, but the same complex conceived at an N/P ratio of 0.5 or 1 showed cytotoxicity when compared to the control (** *p* < 0.01 for the complex at N/P of 0.5 and **** *p* < 0.0001 for the complex at N/P of 1 versus the positive control). Moreover, for all N/P ratio complexes, the viability of U-87 cells decreased with the time of incubation (Figure S5).

This study gave the first insights into the potential effect of the developed complexes on the inhibition of glioma cell viability. It also instigates further in vitro studies to evaluate their therapeutic action, focused on the investigation of the relevance of the co-delivery strategy for enhanced outcomes. If in vitro research reveals itself as promising, the project should include in vivo studies. In this sense, orthotopic glioma-bearing mice models will be constructed, and pharmacodynamic analysis will then be performed. Additionally, frozen brain sections will be examined by fluorescence microscopy and apoptosis will be monitored. This set of studies will certainly bring advances in the targeted delivery protocols aiming at glioblastoma therapy, improving therapeutic efficacy and, therefore, clinical translation.

## 4. Conclusions

Glioblastoma is considered to be the most malignant and aggressive brain tumor. Although significant advances have been made toward glioblastoma therapy, it remains the deadliest human cancer. Nowadays, non-viral cancer gene therapy emerged as a potent and effective therapy to improve the survival rate/life expectancy of cancer patients. Following this, the development of an efficient targeted drug/gene co-delivery system directed to glioma cells is crucial. To meet this goal, our team developed complexes based on the electrostatic and hydrophobic interactions between a cell-penetrating peptide (WRAP5) bearing a targeting sequence, and a tumor suppressor p53-encoding pDNA. To increment the therapeutic potential of the nanocomplexes, the drug TMZ was also incorporated into the formulations. The TMZ/peptide/pDNA complexes exhibited a set of suitable properties for cellular internalization and payload delivery. We found that these characteristics were dependent on the nitrogen to phosphate groups (N/P) ratio and can be optimized to tailor cellular transfection efficiency. A confocal microscopy study showed that the developed complexes were internalized into glioma cells with nucleus localization. Moreover, the p53 gene was expressed and the protein was produced. From this, a considerable cellular viability inhibition was observed, mediated, in particular, by TMZ/Tf-WRAP5/pDNA complexes at an N/P ratio of 1, revealing their promising therapeutic potential. These data prompt additional research on their therapeutic action against glioblastoma cells. Moreover, future studies should include experiments using p53-mutated cells. This work represents a great contribution to the design of targeted drug/gene peptide delivery systems for efficient glioblastoma therapy.

## Figures and Tables

**Table 1:** TMZ-loaded peptides efficiency.

Peptide	TMZ Loading Efficiency (%)
TMZ/WRAP5	60.1 ± 4.8
TMZ/Tf-WRAP5	66.4 ± 8.3

**Table 2:** Mean size, polydispersity index, average zeta potential, and pDNA complexation capacity (CC) for WRAP5/pDNA, Tf-WRAP5/pDNA, TMZ/WRAP5/pDNA, and TMZ/Tf-WRAP5/pDNA complexes formulated at different N/P ratios (0.1, 0.5, and 1). The values were calculated with the data obtained from three independent measurements (mean ± SD, *n* = 3).

System	N/P	Size (nm)	PdI	Zeta Potential (mV)	CC (%)
WRAP5/pDNA	N/P 0.1	398 ± 4	0.447 ± 0.06	+1.49 ± 0.60	51.33 ± 3.06
	N/P 0.5	387 ± 3	0.387 ± 0.03	+3.03 ± 0.29	81.67 ± 2.08
	N/P 1	272 ± 5	0.300 ± 0.03	+4.03 ± 0.11	89.67 ± 4.04
Tf-WRAP5/pDNA	N/P 0.1	349 ± 9	0.543 ± 0.03	+1.78 ± 0.51	50.26 ± 1.53
	N/P 0.5	323 ± 5	0.387 ± 0.04	+3.60 ± 0.30	89.33 ± 3.22
	N/P 1	233± 6	0.327 ± 0.03	+4.43 ± 0.05	94.33 ± 0.58
TMZ/WRAP5/pDNA	N/P 0.1	382 ± 5	0.284 ± 0.05	+2.84 ± 0.19	54.56 ± 2.88
	N/P 0.5	318 ± 8	0.276 ± 0.04	+7.85 ± 0.23	74.89 ± 1.90
	N/P 1	179 ± 4	0.410 ± 0.04	+12.55 ± 0.42	92.56 ± 1.81
TMZ/Tf-WRAP5/pDNA	N/P 0.1	392 ± 5	0.399 ± 0.03	+3.93 ± 0.29	52.89 ± 2.15
	N/P 0.5	252 ± 6	0.379 ± 0.04	+7.90 ± 0.22	71.44 ± 2.46
	N/P 1	182.9 ± 4	0.396 ± 0.04	+11.94 ± 0.29	89.56 ± 2.19

## Data Availability

All the results of this study are presented in the manuscript or in the Supplementary Information.

## Supplementary Materials

The following supporting information can be downloaded at: https://www.mdpi.com/article/10.3390/pharmaceutics14102213/s1, Table S1: Information on WRAP5 sequence, total residues, isotopic mass and positive charge; Figure S1: Calibration curve of TMZ (reference standard) obtained by HPLC method; Scheme S1: Illustration of the formation of peptide/pDNA complexes and electrophoretic evaluation of the complexes or the supernatants; Figure S2: Electrophoretic analysis of WRAP5/pDNA, Tf-WRAP5/pDNA, TMZ/WRAP5/pDNA, and TMZ/ Tf-WRAP5/pDNA complexes formulated at an N/P ratio of 1; Figure S3: Agarose gel electrophoresis for WRAP5/pDNA and Tf-WRAP5/pDNA complexes after its incubation with different culture media and after decomplexation from the complexes with SDS; Figure S4: Fluorescence confocal microscopy evaluation of cellular uptake and intracellular co-localization displayed by the complexes after 2 h of transfection; Figure S5: Cellular viability of U-87 cells after 24 h and 48 h of incubation with the developed peptide/pDNA complexes prepared at N/P ratios of 0.1, 0.5, and 1.
