# Supplementary material for: Development of WRAP5 Peptide Complexes for Targeted Drug/Gene Co-Delivery toward Glioblastoma Therapy
Source: Pharmaceutics. 2022 Oct 18;14(10):2213. doi: 10.3390/pharmaceutics14102213 (PMC9607428; doi:10.3390/pharmaceutics14102213)
Supplement: Supplementary file 1 [file pharmaceutics-14-02213-s001.zip › pharmaceutics-1916995-supplementary.pdf]

Supplementary Material

Table S1. Information on WRAP5 sequence, total residues, isotopic mass and positive charges.

Peptide	Peptide sequence	Total residues	Isotopic mass [g/mol]	Positive charges
WRAP5	NH2-LLRLLRWWWRLLRLL-CONH2	15	2104.34	5

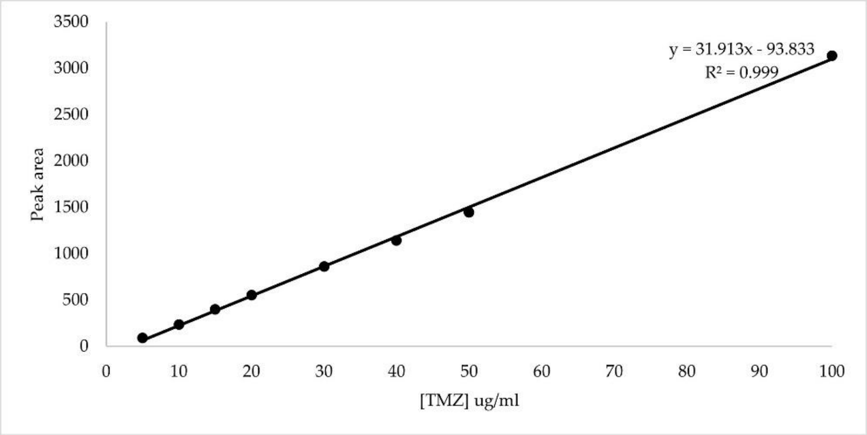

Figure S1. Calibration curve of TMZ (reference standard) obtained by HPLC method.

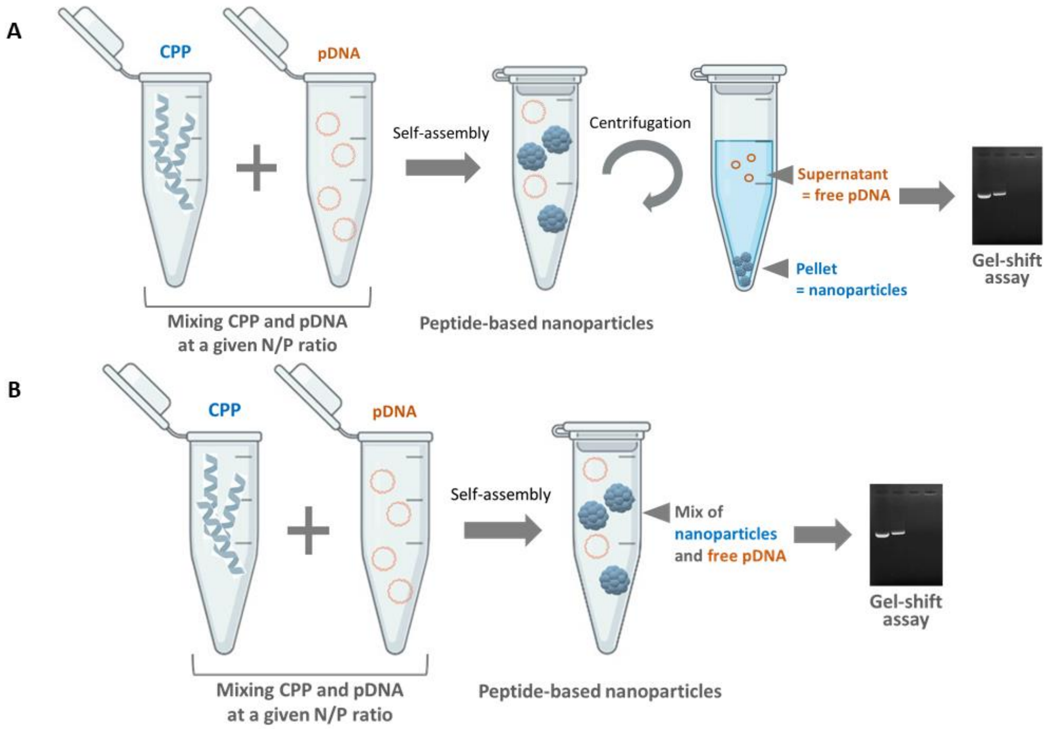

Scheme S1. Illustration of the formation of peptide/pDNA complexes and electrophoretic evaluation of the complexes or the supernatants. The peptide/pDNA complexes were formed by adding peptide solutions to a pDNA solution. Various N/P ratios were considered for complexes formation, ranging from 0.1-5. **A** - The complexes were centrifuged, the pellet containing the complexes was recovered, and to monitor the pDNA complexation capacity (CC) of the developed complexes, only the supernatants were evaluated by agarose gel electrophoresis. **B** – The complexes were formed during 25 min and then directly evaluated by agarose gel electrophoresis assay, without the centrifugation step, to determine for example their stability. Both schemes are partially created using icons of BioRender.com.

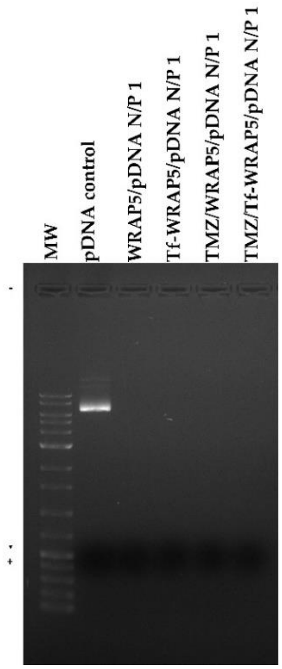

Figure S2. Electrophoretic analysis of WRAP5/pDNA, Tf-WRAP5/pDNA, TMZ/WRAP5/pDNA and TMZ/ Tf-WRAP5/pDNA complexes formulated at N/P ratio of 1. Complexes were loaded on the agarose gel without a centrifugation step (See Scheme S1B) MW – molecular weight ladder.

A

A1

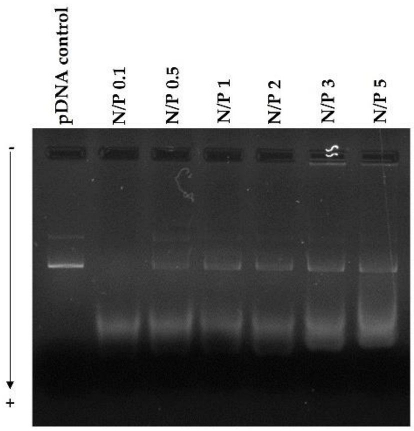

A2

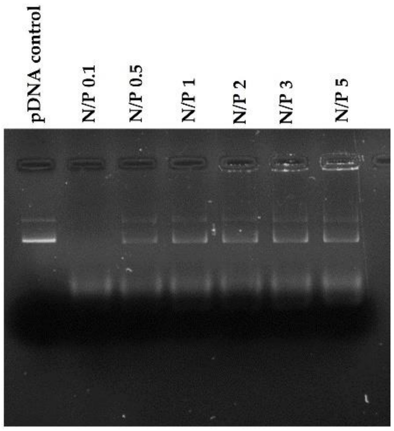

B

B1

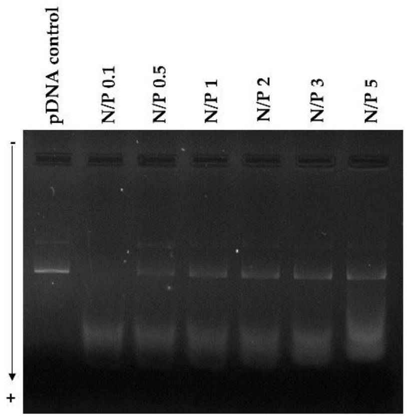

B2

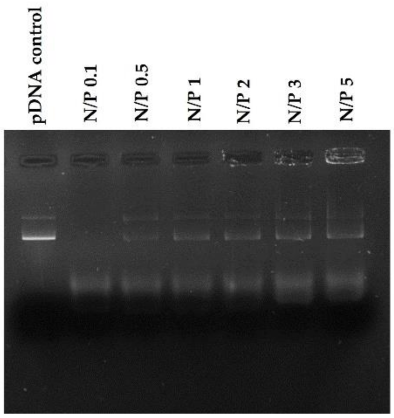

Figure S3. Agarose gel electrophoresis for a set of WRAP5/pDNA (**A**) and Tf-WRAP5/pDNA (**B**) complexes formulated at N/P ratios of 0.1, 0.5, 1, 2, 3 and 5, after its incubation with DMEM/Nutrient Mixture F-12 Ham (DMEM/F-12) with L-glutamine (1) and DMEM high glucose with stable glutamine medium (2) and after decomplexation from the complexes with 10 % SDS. Complexes were loaded on the gel without a centrifugation step (See Scheme S1B). Lane 1: pDNA control.

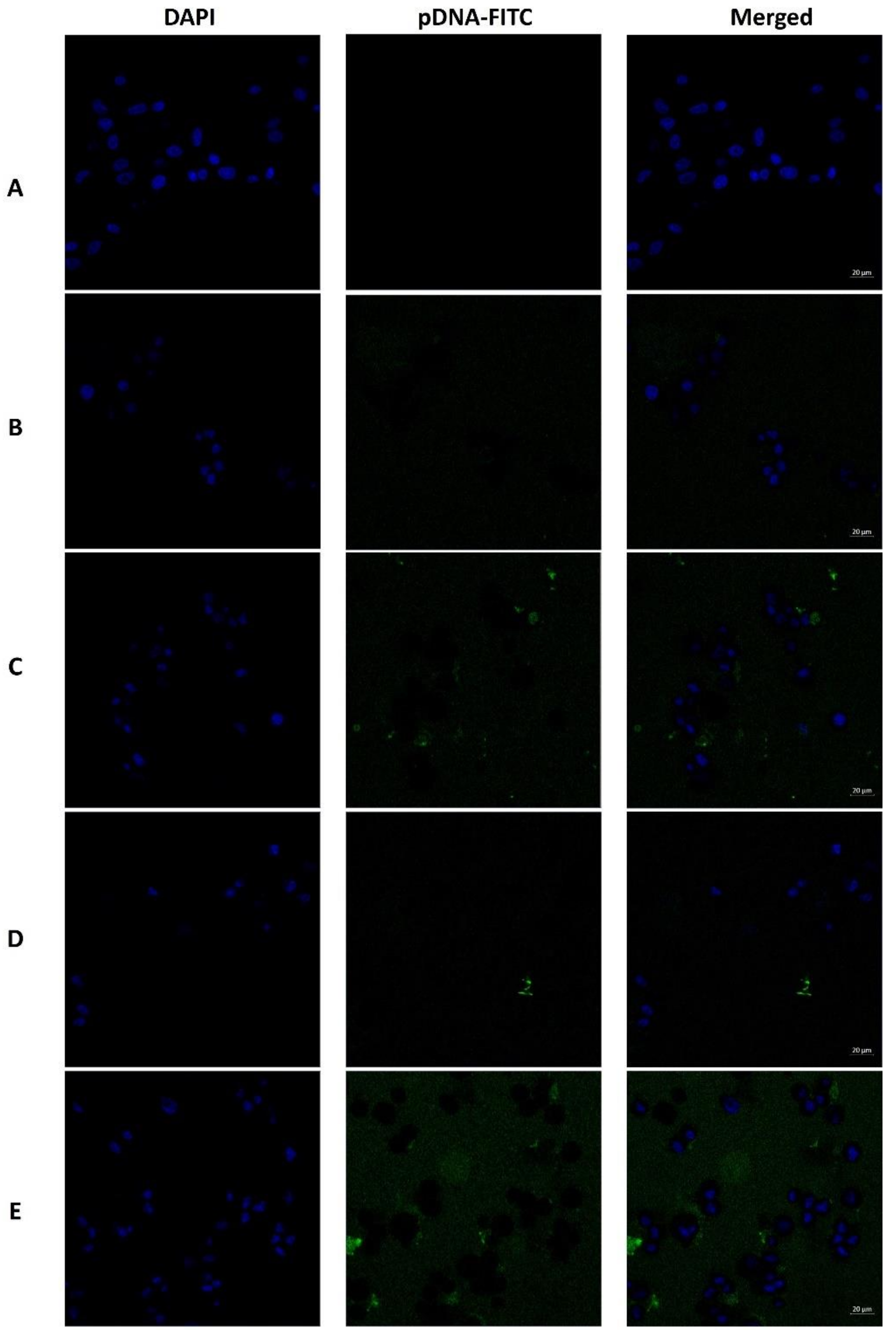

Figure S4. Fluorescence confocal microscopy evaluation of cellular uptake and intracellular co-localization displayed by the set of complexes prepared at N/P ratio of 1 after 2 h of transfection. Nuclei were stained by DAPI and green represents pDNA labeled by FITC. Representative live-cell images of non-transfected U-87 cells (A), and U-87 cells transfected with the following complexes: WRAP5/pDNA (B), Tf-WRAP5/pDNA (C), TMZ-WRAP5/pDNA (D), TMZ-Tf-WRAP5/pDNA (E). Scale bar = 20 μm.

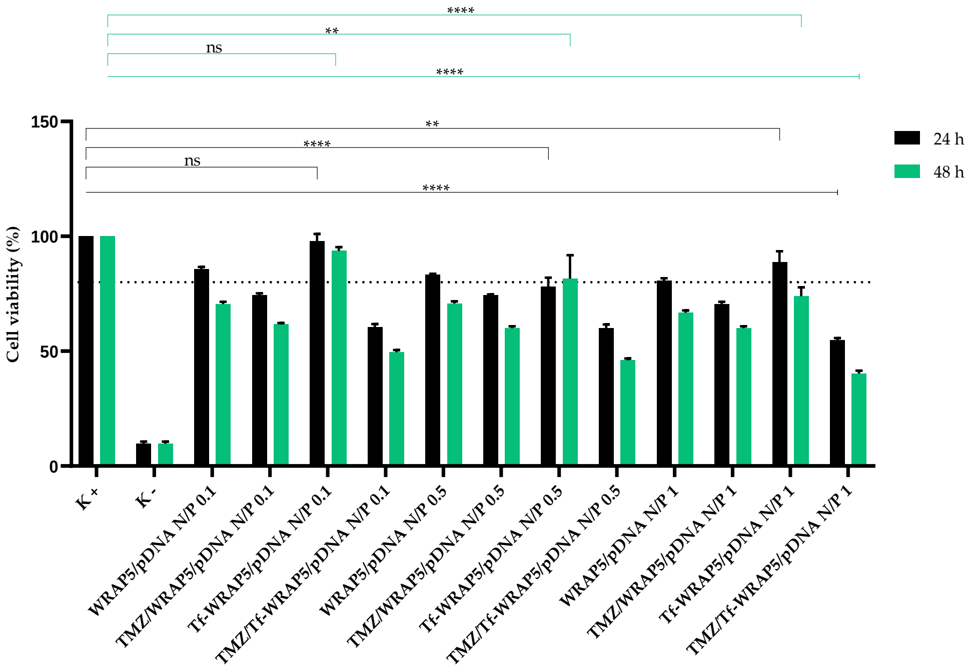

Figure S5. Cellular viability of U-87 cells after 24 h and 48 h of incubation with WRAP5/pDNA, TMZ/WRAP5/pDNA, Tf-WRAP5/pDNA and TMZ/Tf-WRAP5/pDNA complexes prepared at N/P ratios of 0.1, 0.5, and 1. Non-transfected cells were used as positive control (K+) and cells treated with ethanol were used as negative control (K-). Statistical analysis was made using one-way ANOVA with data obtained from four independent measurements (mean ± SD, n = 4). (****$p \leq 0.0001$; **$p \leq 0.01$).
